# Intracranial melanocytic meningeal tumours and melanosis oculi: case report and literature review

**DOI:** 10.1186/1471-2407-12-220

**Published:** 2012-06-06

**Authors:** Francesco Doglietto, Cesare Colosimo, Libero Lauriola, Mario Balducci, Pasquale De Bonis, Nicola Montano, Gelareh Zadeh, Giulio Maira, Roberto Pallini

**Affiliations:** 1 Institute of Neurosurgery, Catholic University School of Medicine, Rome, Italy; 2 Institute of Radiology, Catholic University School of Medicine, Rome, Italy; 3 Institute of Pathology, Catholic University School of Medicine, Rome, Italy; 4 Institute of Radiotherapy, Catholic University School of Medicine, Rome, Italy; 5 Division of Neurosurgery, Toronto Western Hospital, Toronto, ON, Canada; 6 Catholic University School of Medicine, Institute of Neurosurgery, Largo A. Gemelli, 8 00168, Roma, Italy

**Keywords:** Melanocytoma, Melanocytic meningeal tumour, Intracranial melanocytosis, Melanosis oculi, Cavernous sinus

## Abstract

**Background:**

Melanocytic meningeal tumours are rare extra-axial neoplasms of the nervous system, with only three reported cases in the cavernous sinus. Herein we describe for the first time the association of ocular melanosis and multiple intracranial melanocytic meningeal tumours, with the presenting lesion being in the cavernous sinus. The importance of this association is discussed together with the diagnostic and therapeutic challenges of the case.

**Case presentation:**

A 20-year-old man presented with a left sixth cranial nerve deficit; general examination documented only congenital melanosis of the homolateral eye. MRI examination showed a space occupying lesion in the left cavernous sinus, which was followed conservatively for 2 years, until a new space occupying lesion was evident at the level of the right frontal convexity: both lesions presented with neuroradiological characteristics suggestive of melanin content.

The frontal convexity lesion was removed: intraoperatively the dura was markedly and diffusely melanotic. Histological examination documented a melanocytic meningeal tumour, with a proliferative index of 3 %. The patient underwent 3D-Conformal Radiation Therapy on the lesion of the cavernous sinus (total dose 5040 cGy), with initial tumour reduction. Three years later, due to a symptomatic growth, he underwent partial removal of the lesion in the cavernous sinus. Histological examination was unchanged. He then received adjuvant Temozolomide with Low Dose Fractionated Radiation Therapy (LD-FRT). Due to further disease progression cisplatin plus fotemustine were administered, concomitant with LD-FRT: after two cycles MRI documented significant disease regression. After a period of apparent disease control, the patient presented with persistent cough and evidence of multiple thoracic metastases, which lead to his death, seven years after presentation.

**Conclusions:**

Intracranial melanocytic meningeal tumours are challenging lesions, both from a diagnostic and therapeutic point of view; though rare, the possible association with ocular melanosis should be recognized and might facilitate an early diagnosis. Surgery remains the best possible option when feasible. In the event of partial resection, this “benign” disease might be clinically aggressive.

## Background

Melanocytic meningeal tumour is a rare tumour of the nervous system with approximately 110 reported cases [1-3]. The possible association with nevus of Ota (also known as oculo-dermal melanocytosis, which is characterized by the association of ocular and skin or mucosal melanocytosis) [2,4-9] has been reported previously.

Herein we describe the unique case of the association of simple ocular melanosis and multiple intracranial melanocytic meningeal tumours. The diagnostic and therapeutic challenges of the case are presented together with a concise literature review.

## Case report

### Clinical and neuroradiological presentation

**Figure 1:**
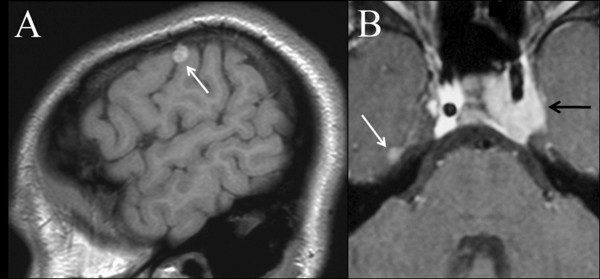
**Brain MRI. A) **Unenhanced turbo-spin-echo T1-weighted sagittal image documenting the space occupying lesion at the level of the left frontal convexity (white arrow).**B) **Contrast-enhanced fat-suppressed turbo-spin-echo T1-weighted axial image**. **MRI documenting the space occupying lesions at the level of the right petrous apex (white arrow) and left cavernous sinus (black arrow).

A 20 year-old Caucasian male presented to our department with incomplete left abducens nerve palsy; neuroradiological examination documented three intracranial space occupying lesions, respectively in the left cavernous sinus, on the right frontal convexity and on the superior aspect of the right petrous pyramid (Figure 1).

**Figure 2:**
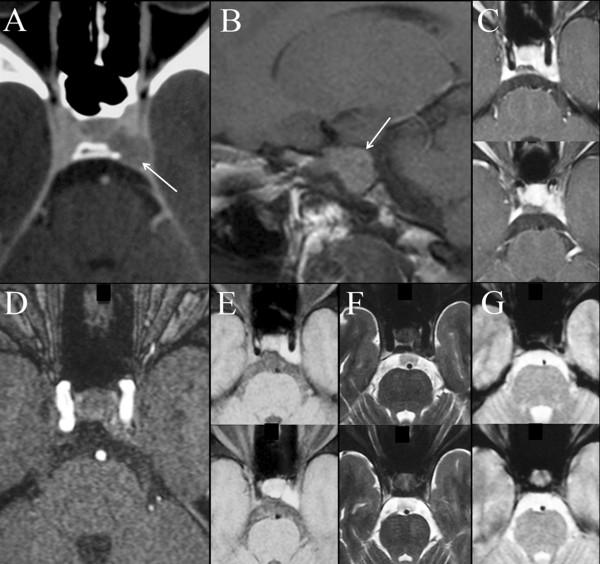
**Neuroradiological findings at diagnosis of the lesion inside the cavernous sinus. **Contrast enhanced CT **(A), **documenting the space occupying lesion in the left cavernous sinus. T1-weighted images documenting the lesion, which is spontaneously hyper-intense (sagittal view; **B **– white arrow) and has scarce contrast-enhancement (fat suppressed turbo spin echo T1-weighted axial images after contrast enhancement; **C). **Axial source image from MR-angio ruled out a vascular lesion **(D). **The mass lesion remained hyper-intense on un-enhanced fat-saturation T1-weighted images **(E), **strongly hypo-intense on turbo-spin echo fat-saturated T2-weighted images **(F), **and iso-intense on gradient echo T2*-weighted images **(G).**

He presented with a two year history of diplopia, of subacute onset, due to a left sixth cranial nerve palsy. Neurological examination was otherwise normal at the time; he denied any pain and had no fever. Head CT scan documented a small space-occupying lesion in the left cavernous sinus, with well-defined margins and minimal contrast enhancement (Figure 2A). Brain MRI confirmed the lesion in the posterior compartment of the left cavernous sinus: its signal behaviour excluded both fatty and hemorrhagic content of the tumour, excluding the hypothesis of paramagnetic effect related to hemosiderin and blood degradation products (Figure 2). Due to the high complication rate associate with surgery inside the cavernous sinus, specifically with respect to a very high risk of permanent diplopia, his treating doctors chose to manage him conservatively with low dose corticosteroids and surveillance imaging. His diplopia resolved and a new MRI, three months after the previous, documented a minimal volume reduction of the left cavernous sinus mass lesion. Unfortunately the patient experienced bilateral femoral head necrosis secondary to corticosteroid use that resulted in discontinuation of dexamethasone. Three months later a new brain MRI documented minimal growth of the lesion, which was confirmed in subsequent examinations and the patient remained asymptomatic. One year and a half after the first neuroradiological examination, a repeat brain MRI documented a contralateral extraaxial lesion, at the level of the right frontal convexity, with the same radiological features, characterized by hyper-intensity on un-enhanced T1-weighted images (Figure 1A); a smaller extraaxial dural lesion was also evident on the superior aspect of the right petrous pyramid (Figure 1B).

**Figure 3:**
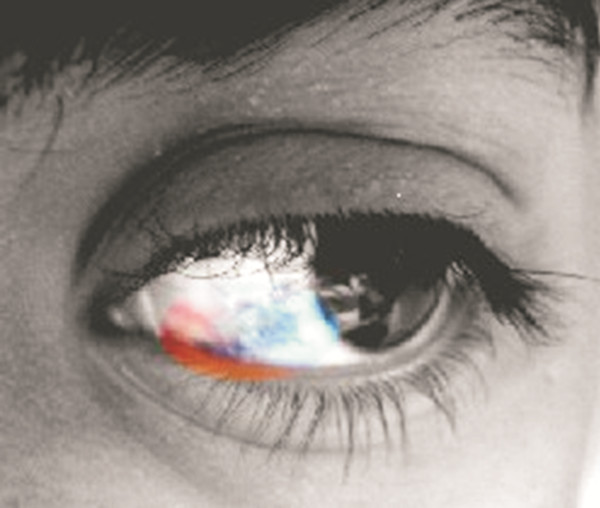
**Melanosis oculi. **Black and white picture of the left eye: the part of the sclera involved by the bluish discoloration has been left in the original color.

**Figure 4:**
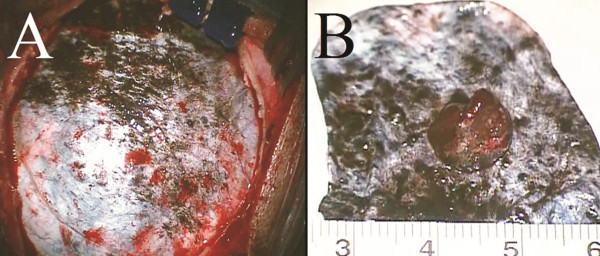
**Intraoperative findings at removal of the frontal lesion. (A.) **Diffuse blackening of the dura was evident with an increasing gradient towards the skull base. **(B.) **The removed nodule attached to and surrounded by dark dura.

At this stage in his care the patient was referred to our department. Neurological examination documented diplopia only in the extreme left gaze. General examination documented patches of bluish discoloration of the sclera at the level of the left eye, which the mother reported to be congenital (Figure 3); there was no evidence of any associated facial skin or oral mucosal pigmentation. The association of the bluish discoloration of the sclera with the MR signal behaviour (non-hemorrhagic T1-hyper-intensity) of the intracranial lesions raised the suspicion of a melanotic tumour, and our opinion was to offer surgery in order to establish definitive diagnosis; it was decided to remove the frontal lesion, having the least surgical risk. After a right frontal craniotomy diffuse blackening of the dura was evident with a darker grading towards the skull base (Figure 4A); the nodular lesion appeared to partially infiltrate the arachnoid and was totally removed together with portion of the surrounding abnormal dura (Figure 4B).

### Histology

**Figure 5:**
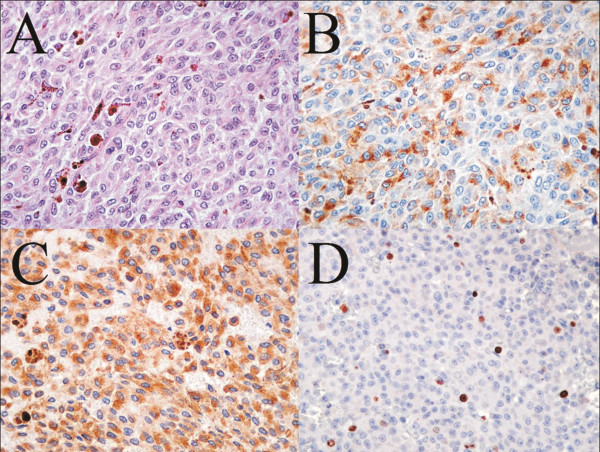
**Histological examination. **Haematoxylin and eosin staining **(A **- original magnification: X 300) documenting round or spindled neoplastic cells, with regular nuclei and small nucleoli, with only occasional mitoses (less than 1x10 HPF); melanin pigment is evident in occasional cells. Immunohistochemistry with anti-HMB45 **(B) **and anti Melan-A **(C) **antibodies documented a variable but substantial proportion of labelled neoplastic cells (original magnification: X 300). MIB1/Ki67 immunohistochemistry **(D) **documented only a low percentage of labelled neoplastic cells (mean: 3 %) (original magnification: X 250).

On haematoxylin and eosin (H&E) staining (Figure 5A – original magnification: X 300) neoplastic cells appeared round or spindled, without nuclear atypia and with small nucleoli. There were occasional mitoses identified (less than 1x10 HPF). Melanin pigment was present in occasional cells, so we were able to study the nuclear morphology without the need of a melanin bleaching. No necrosis was observed. After immunohistochemistry with anti-HMB45 (Figure 5B) and anti Melan-A (Figure 5C) antibodies, a variable but substantial proportion of neoplastic cells appeared immunolabelled (original magnification: X 300). After immunohistochemistry with anti MIB1/Ki67 antibody (Figure 4D) only a low percentage of neoplastic cells (mean: 3 %) showed nuclear labelling (original magnification: X 250). On the basis of the single small nucleoli, the relatively low number of mitoses, and the relatively low Ki67 index, a diagnosis of intermediate grade melanocytic neoplasm of was made [10]. Moreover, all the histological features we encountered in our case but Ki67 were also compatible with a melanocytoma.

### Follow-up

Five days after surgery the patient developed a marked diplopia, pain in the eye and a third cranial nerve palsy. An urgent CT scan documented a minimal enlargement of the cavernous sinus lesion (not shown). Corticosteroid treatment was initiated and three weeks later there was complete resolution of both pain and cranial nerve palsy. Corticosteroids were subsequently tapered and discontinued. The patient underwent 3D-Conformal Radiation Therapy (3D-CRT) of the lesion of the cavernous sinus (total dose 5040 cGy with a daily dose of 180 cGy), with an initial tumour reduction. Three years later, due to a symptomatic growth, the patient underwent surgical debulking of the lesion with resolution of the pre-operative trigeminal neuralgia and persistence of third cranial nerve palsy. Histological examination of the lesion documented features similar to the previously resected lesion, with a proliferative index of 2 %.

He then received four cycles of adjuvant Temozolomide (150–200 mg/mq/die for 5 days q 28); the third and fourth cycle were combined with Low Dose Fractionated Radiation Therapy (LD-FRT) 0.4 Gy twice daily [11,12], with at least a 6-hour inter-fraction interval, over 5 consecutive days, every 28 days. Subsequent MRI examinations documented progressive disease: we then prescribed cisplatin (30 mg/m2 on days 1, 8, and 15) and fotemustine (40 mg/m2 on days 2, 9, and 16) [13], concomitant with LD-FRT 0.3 Gy twice daily, with at least a 6-hour inter-fraction interval [11] on days 1–2, 8–9, and 15–16, every 42, with at least a 6-hour inter-fraction interval, over 5 consecutive days, every 28 days. After two cycles MRI documented significant tumour reduction and there was a period of apparent tumour control. Unfortunately, the patient later presented with persistent cough and hypoxia: a total body CT scan documented multiple thoracic space occupying lesions, compatible with metastases. The patient died due to systemic tumour progression seven years after initial presentation.

## Discussions

Meningeal melanocytic tumour is a rare tumour of the nervous system, with approximately 110 cases reported since 1972 [1,5]. It originates from melanocytic cells located in the leptomeninges and is usually a solitary lesion, mostly located in the posterior fossa and along the cervico-thoracic spinal cord [14]. To the best of our knowledge, only three case of melanocytoma of the cavernous sinus have been described [8,15,16]. According to Brat [10], primary melanocytic neoplasms consist of a spectrum of tumours ranging from melanocytomas to malignant melanomas. Melanocytic tumours with intermediate features (intermediate grade melanocytic neoplasms) share almost all the histological features of melanocytomas and most of the features of malignant melanomas. Below we identify some factors that render these tumors difficult to diagnose and their biological behaviour challenging to predict.

### Neuroradiology

On computerized tomography (CT) imaging, melanocytic tumors appear as iso- to slightly hyper-dense lesions with homogeneous enhancement after contrast media administration [17,18]. This CT appearance is though non-specific and similar to that of meningiomas. On MRI melanocytic tumors are generally reported as iso- to hyper-intense on T1-weighted images and hypo-intense on T2-weighted images, with variable degree of homogeneous enhancement with paramagnetic contrast injection [17-21]. Unfortunately MRI appearance of melanocytic tumours may vary due to their heterogeneous content of melanin pigment as well as the presence of other features, such as intratumoral hemorrhages. Thus, the classically reported neuro-imaging features could not reliably distinguish melanocytic tumors from other most common tumors that may be found in similar dural locations, i.e., most notably meningioma, schwannoma and malignant melanoma. [18] However on the basis of our personal experience (4 pathologically proven, mostly intraspinal, melanocytic tumors – C. Colosimo, MD, unpublished data, April 2009) the rational analysis of the MR signal behaviour strongly suggests a melanocytic tumors when the lesion appears hyper-intense on un-enhanced T1-weighted images, at the same time showing less intensity on gradient-echo T2*-weighted than on turbo spin echo T2-weighed images.

### Melanocytic tumors and melanosis

Intracranial melanocytic non-metastatic tumors can be associated with nevus of Ota or oculodermal melanosis [4]; to the best of our knowledge, the association of melanocytic tumors with simple melanosis oculi [22] has not been reported: only one case of intracranial melanoma associated with melanosis oculi has been previously described [23]. The importance of this association cannot be overestimated for the clinical differential diagnosis. From an ethiopathogenetic point of view the presence of congenital melanosis oculi and the intraoperative finding of diffuse blackening of the dura are suggestive of an evolution of a congenital malformation to cancer. It has indeed been suggested that meningeal melanosis, a benign excess of melanotic cells in the leptomeninges, may be a predisposing lesion that could transform into melanocytic tumor or CNS melanoma [6,8].

### Treatment

Complete surgical resection is the best treatment for meningeal melanocytic tumors [1]. If not possible, partial tumor removal followed by local radiotherapy is suggested [1]. Primary radiotherapy or radiosurgery may be useful for tumors, which are not amenable to complete surgical resection, as the cavernous sinus lesions in our case [24].

Radiotherapy improves outcomes and a total dose of 50.4 Gy (1.8 Gy per fraction) appears appropriate for most patients [25]. Radiosurgery may be an alternative therapeutic option, but specific reports are still limited [24].

To the best of our knowledge there is also a lack of data concerning the role of chemotherapy in the treatment of melanocytic tumors [26]. Our decision to prescribe chemotherapy was related to the disease progression after radiation therapy and incomplete resection. Temozolomide, an oral alkylating agent, and Fotemustine (FTM), a third-generation nitrosourea with an alkylating cytotoxic activity, were used because they are able to cross the blood–brain barrier; moreover their efficacy in treating newly diagnosed glioma or melanoma brain metastases has been largely demonstrated in combination with radiotherapy.

Rare cases have been reported with leptomeningeal spread [27-29] transformation to malignant melanoma [30] and even distant metastases [31-34]. This potentially aggressive behaviour led Cordoba and co-workers [35] to suggest that melanocytic tumors should be considered as a borderline malignant tumour. This is indeed confirmed by the clinical evolution of our patient, who had an apparently stable disease six years after initial symptoms and diagnosis, but only after chemotherapy and radiotherapy; unfortunately, his disease further progressed, with evidence of systemic metastases that lead to his death. This supports the use of the term "melanocytic neoplasm of indeterminate biologic potential" for tumours that demonstrate intermediate histological features and might have an aggressive clinical behavior [36].

## Conclusions

This is the first reported case of the association of simple ocular melanosis and intracranial melanocytoma. Awareness of this association and of the radiological characteristics of the lesion might help the pre-operative diagnosis of this rare intracranial tumor. The clinical course of our patient indeed confirms the relatively aggressive behavior of this tumor.

## Consent

Written informed consent was obtained from the patient for publication of this Case report and any accompanying images. A copy of the written consent is available for review by the Series Editor of this journal.

## Competing interests

The authors have no competing interests.

## Authors’ contributions

FD conceived the study, reviewed the literature and wrote the first draft; CC conceived the study, provided the radiological data, wrote the draft of the radiological part of the study and critically reviewed the manuscript; LL provided the histological data, and critically reviewed the manuscript; MB provided the data on the radiotherapic treatment, and critically reviewed the manuscript; PDB conceived the study, reviewed the literature and critically reviewed the manuscript; NM reviewed the literature and critically reviewed the manuscript; GZ critically reviewed the drafts and final manuscript; GM critically reviewed the manuscript; RP conceived the study and critically reviewed the manuscript. All authors read and approved the final manuscript.

## Pre-publication history

The pre-publication history for this paper can be accessed here:

http://www.biomedcentral.com/1471-2407/12/220/prepub
